# The dawn of physiological closed-loop ventilation—a review

**DOI:** 10.1186/s13054-020-2810-1

**Published:** 2020-03-29

**Authors:** Philip von Platen, Anake Pomprapa, Burkhard Lachmann, Steffen Leonhardt

**Affiliations:** 1 Medical Information Technology, Helmholtz-Institute for Biomedical Engineering, RWTH Aachen University, Pauwelsstr. 20, Aachen, 52074 Germany; 2 Department of Anesthesiology and Operative Intensive Care Medicine (CCM, CVK), Charité - Universitätsmedizin Berlin, corporate member of Freie Universität Berlin, Humboldt-Universität zu Berlin, and Berlin Institute of Health, Augustenburger Platz 1, Berlin, 13353 Germany

**Keywords:** Closed-loop ventilation, Patient-in-the-loop, Physiological control

## Abstract

The level of automation in mechanical ventilation has been steadily increasing over the last few decades. There has recently been renewed interest in physiological closed-loop control of ventilation. The development of these systems has followed a similar path to that of manual clinical ventilation, starting with ensuring optimal gas exchange and shifting to the prevention of ventilator-induced lung injury. Systems currently aim to encompass both aspects, and early commercial systems are appearing. These developments remain unknown to many clinicians and, hence, limit their adoption into the clinical environment. This review shows the evolution of the physiological closed-loop control of mechanical ventilation.

## Introduction

Closed-loop systems are designed to dynamically regulate a given variable around a desired set point. Examples thereof surround our everyday lives, from cruise-control maintaining the correct speed on the highway, to auto-pilot flying modern airplanes safely.

Modern medical systems are increasingly incorporating these technological advances. Medical applications of closed-loop control can be divided into systems that control a physical variable of the medical device and those that control a physiological variable of the patient [1]. Many medical devices already incorporate *device-internal* closed-loop control systems. An example is the internal regulation of the fraction of inspired oxygen to the value set by the clinician. If there is some backward interaction between the patient and the medical device, the system can be said to be *patient-oriented*. The control of airway pressure during pressure-controlled ventilation is such an example, because there is interaction between the device and patient. Here, the controller also focuses on the medical device.

Regulating a physiological variable of the patient is known as physiological closed-loop control (PCLC). With the extensive physiological monitoring in today’s clinical environment, PCLC is becoming ever more popular. In this case, the patient is the focus of the control loop and a physiological measurement is fed back to the controller. The PCLC has recently been taken up by regulatory bodies, with an international standard developed specifically for it, namely the IEC 60601-1-10 [2] and a public workshop held by the Food and Drug Administration in 2015 with subsequent publication by Parvinian and colleagues in 2018 [3].

Importantly, PCLC allows for the automation of certain therapeutic tasks currently performed by medical staff. Critical and emergency care especially is presumed to benefit from increased automation, as these high-stress environments have a shrinking workforce, as projected by Angus et al. [4]. This limited supply of medical staff, coupled with the labor-intensive practice of setting a ventilator correctly [5, 6] means that proper, personalized patient care will become even more difficult in the future. The already high costs of keeping patients on mechanical ventilation [7, 8] will also increase even further. If some ventilator settings are adjusted automatically, this would increase the time and capacity of the medical staff.

Despite regaining attention recently, PCLC of mechanical ventilation has been around for over half a century. Similar to the guidelines of mechanical ventilation which have evolved over time, from focusing on optimal gas exchange to reducing ventilator-induced lung injury [9], a similar trend can be seen in the research into PCLC for mechanical ventilation. This review aims to show the evolution of the PCLC of mechanical ventilation and its close relationship with the clinical goals of mechanical ventilation.

## Scope and definitions

The criteria of search for this paper was as follows. Firstly, relevant literature was identified from database search combinations of the keywords: closed-loop, control, feedback and automation in combination with ventilation, mechanical ventilation, and artificial ventilation. Secondly, the search was narrowed down to include only systems which used patient-specific physiological variables for the feedback control. The physiological variables can be grouped loosely into oxygen, carbon dioxide, respiratory mechanics, and patient demand. Thirdly, the phase of weaning has so far benefited most from automation and was therefore added as an additional search keyword. Finally, only literature including studies with patients or medium/large animals was included. Extensive additional literature on theoretical approaches and simulative results exists, but these still need to be validated experimentally.

Furthermore, previous reviews on closed-loop mechanical ventilation exist and provided further relevant literature. Brunner presented an important early manuscript describing the history and principles of closed-loop ventilation [10]. Reviews of advanced closed-loop control in mechanical ventilation have been provided by Lellouche et al. [11], Wysocki et al. [12], and Branson [13].

In order to present a precise review, ventilation modes and breathing patterns are not discussed; these have been covered elsewhere, (e.g., Chatbrun et al. [14]). In addition, other methods of ventilation, such as liquid, noisy, and high-frequency ventilation, are beyond the scope of this paper.

## Concept of physiological closed-loop ventilation

The primary goal of mechanical ventilation is to rectify and maintain adequate gas exchange. Ventilating patients protectively has become another important goal during mechanical ventilation.

The current clinical practice for choosing ventilator settings is very complex and based on expert knowledge. The modern clinician relies on classical physiologic variables for mechanical ventilation, such as peripheral capillary oxygen saturation (SpO_2_), end-tidal CO_2_ (etCO_2_), and blood gas analysis (PaO_2_, PaCO_2_, pH). In addition, clinicians consider the protective guidelines, hemodynamic parameters (heart rate, blood pressure, perfusion), and derived variables such as the pF ratio, the oxygen A-a gradient, shunt, transpulmonary pressure, and mechanical power, among others [9].

**Fig. 1:**
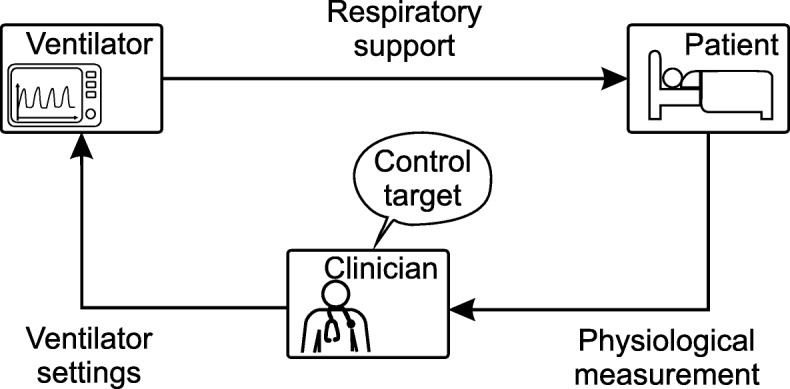
Classical clinician-in-the-loop system. The physiological measurement and ventilator settings shown are only exemplary. In the clinical environment, further derived measurement variables are also used. Clinician refers to the physicians, respiratory therapists, or nurses

The interaction of clinician, ventilator, and patient forms a manual closed-loop system or “clinician-in-the-loop” system. This is shown in a block diagram in Fig. 1. Here the clinician acts as the controller and compares the available physiological and derived measurements to a defined control target before deciding on appropriate ventilator settings (actuation).

This clinician-in-the-loop system is labor-intensive and time-consuming, as the presence of the clinician is always necessary. The clinician’s full attention is required to adjust ventilator settings if the patient state changes and to accommodate new therapeutic needs. If the clinician is not present, the system becomes an open-loop system, which is unable to respond if the oxygenation or ventilation become insufficient due to worsening patient conditions or external disturbances. This may lead to the patient being poorly and not protectively ventilated, with possible dire consequences.

### Characteristics of closed-loop ventilation

**Fig. 2:**
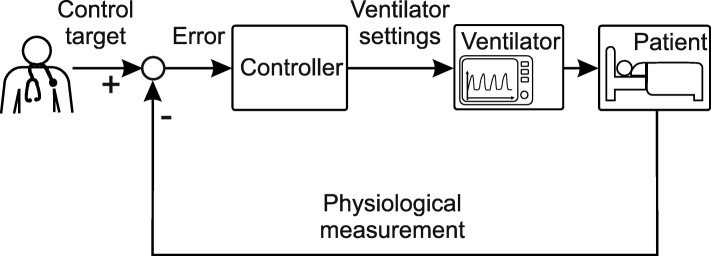
Physiological closed-loop control for mechanical ventilation system

An automated closed-loop system (also known as feedback control) can be implemented to keep a patient at a specified target and respond to disturbances without the clinician’s presence being necessary. Hereby, a controller takes over the task of adapting ventilator settings. The new control loop is depicted in Fig. 2 and shows the subtraction of a measured value from the target value and the error being fed into the controller. The controller then automatically decides on the correct ventilator settings to minimize the error.

Importantly, the clinician’s focus changes to choosing personalized targets, regulating variables supplementary to the ventilation, such as hemodynamics and fluids, and monitoring the system. It should be noted that the current PCLC systems limit their measurement data to classical ventilation variables, as will be shown later. Derived evaluations, such as pF ratio, stress and strain, or heart-lung interactions, have not yet been used.

**Fig. 3:**
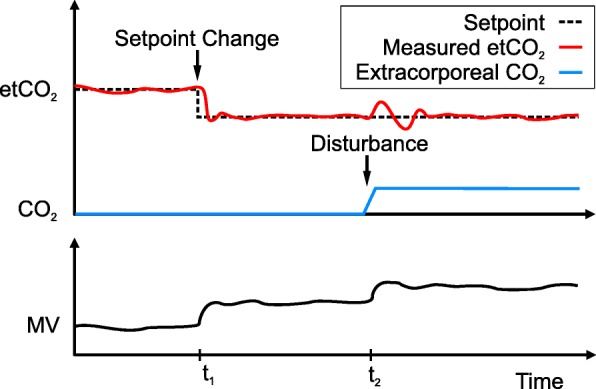
Setpoint tracking and disturbance rejection shown for an illustrative example. A good controller ensures that the measured etCO_2_ closely follows the setpoint. At *t*_1_, a setpoint change (change in target) requires an increase in minute volume (bottom graph). At *t*_2_, a sudden increase in CO_2_ (disturbance) requires another increase in MV

The goals of an automated closed-loop system can be divided into setpoint tracking and disturbance rejection. For most cases, setpoint control is only relevant when the controller is switched on, as it describes the dynamic response of the system until the target is reached. Setpoint changes are rare in the clinical environment. Exemplarily, the SpO_2_ target may be at 88–95% and seldom changes. The disturbance rejection, however, is the true hero during PCLC, as it is concerned with the response of the system to disturbances and bringing the patient back to the target range. Disturbances can take various forms, both internal (worsening illness, lung stiffness, increased CO_2_ production) and external (disconnection, room temperature). The two goals are shown for an illustrative example in Fig. 3, with a setpoint change at *t*_1_ and a disturbance (extreme increase in CO_2_) at *t*_2_.

The evolution of automated physiological control can be categorized by two drivers: the control target and the controller design. The control target is dictated on the one hand by the measurements and sensors available, some of which have been described above. On the other hand, changing clinical evidence and guidelines present new control targets. The second driver, controller design, remains an engineering problem and is beyond the scope of this paper. A brief overview is given here only to facilitate understanding. A natural evolution of the complexity of the controllers comes with the use of computers and their increasing computing power, and the detailed modeling of the respiratory system, which is used for controller synthesis and testing. Simple proportional, integral, and derivative (PID) controllers were used initially [15, 16]. This summation of PID action is the most common controller used in almost all industries nowadays. The tuning (setting up of this controller) is static, meaning the controller needs to be tuned to every patient. To overcome this, adaptive controllers [17–19] can be used, which change their controller parameters to adapt to the patient or scenario. A mathematical model of the system is required for this, but recent advances in modeling respiratory systems have allowed their increased use. The optimal controller attempts to solve an optimization problem, such as minimizing a cost function, in calculating the actuation variable [20]. Model predictive control (MPC) is another example of an advanced controller which uses a dynamic model of the system [21, 22]. It optimizes the current ventilator settings for the current state, while anticipating future events. Finally, there has also been increasing use of fuzzy control [23, 24]. A fuzzy controller is a rule-based control law, in which the linguistic rules (e.g., if-then-else rules) are fuzzified (blurred) in order to make the control more elastic. These systems are popular in the clinical environment, as the language-based expert knowledge of clinicians is transferred into the design of the controller (which is not the case in PID control, for example). More robust controllers may be introduced in the future. These are designed with uncertainty in mind and can control the performance of the system even for the worst case.

## History of physiological closed-loop control

The PCLC of ventilation covers a broad range of control targets. They can be grouped into controllers focusing on gas exchange, lung mechanics (protective ventilation), patient demand, and automation of clinical protocols.

### Control based on gas exchange

The first application of PCLC in mechanical ventilation was presented by Saxton in 1953, with a publication appearing in 1957 [15], where his team applied feedback control to the iron lung to regulate the etCO_2_. In the same decade, Frumin developed an automated anesthesia system, which incorporated an etCO_2_ feedback control system [16, 25]. Both systems used the ventilation pressure as the actuating variable and were able to keep end-tidal CO_2_ at the set target.

In 1971, Mitamura and colleagues controlled the mixed expired CO_2_ using both tidal volume (*V*_*T*_) and breathing frequency (*f*) [20]. Their system was able to keep PCO_2_ at the target, even with extracorporeal CO_2_ loading. Other groups focused on setting either *V*_*T*_ or *f* automatically, with the clinician setting the other variable manually [26, 27]. Digital control using computers for CO_2_ control started with Coles et al. in 1973 [26], and the number of publications about feedback control in ventilation increased. Coles et al. showed that the PCLC system maintained the etCO_2_ at the target better than manual control [26].

The availability of intravascular sensors for pH or PaCO_2_ measurement introduced new closed-loop control systems. Schulz et al. used such a sensor and feedback control to respond to setpoint changes in PaCO_2_ [28]. The response dynamics of their system was, however, limited by the slow response time of the sensor. A similar sensor problem caused the system of Coon et al. [29] to oscillate. The continuous intravascular sensors, however, failed to remain commercially available and their use has ceased. Without any clinical alternatives, the control of CO_2_ was again based on the measurement of etCO_2_. However, the increasing difference between PaCO_2_ and etCO_2_ in the pathological lung requires compensation [20, 28, 30]. Approaches based on first principles to link etCO_2_ and PaCO_2_ were presented by Ohlson et al. [30], but the authors could not compensate correctly when a large variation of cardiac output appeared.

An extensive list of CO_2_ feedback control (often referred to as ventilation control) systems over the past 50 years is shown in Table 1. As can be seen, all systems presented so far have been limited to proof-of-concept studies.

**Table 1:** Chronological development of closed-loop ventilation for CO_2_ and pH control in vivo

Year	First author	Controller type	Patient	Ventilation variables	Subject	Setpoint control	Disturbance control
1957	Saxton [15]	PI	etCO_2_	*P*_*insp*_	Patients (*n* = 2)	x	o
1957	Frumin [16]	PI	etCO_2_	*P*_*insp*_	Patients (*n* = 64)	x	o
1959	Frumin [25]	PI	etCO_2_	*P*_*insp*_	Patients (*n* = 50)	x	o
1968	Holloman [31]	PI	etCO_2_	FiCO_2_	Patient (*n* = 1)	x	o
1971	Mitamura [20]	Optimal	$\dot {\mathrm {V}}\text {CO}_{2}$	*V*_*T*_, *f*	Dogs (*n* = 18)	x	x
1973	Coles [26]	PI	etCO_2_	*V*_*T*_	Sheep (*n* = 1)	x	o
1974	Schulz [28]	PD	PaCO_2_	*V*_*T*_	Patients (*n* = 11)	x	o
1978	Coon [29]	PID	pH	*V*_*T*_	Dogs (*n* = 30)	x	x
1978	Smith [27]	PI	etCO_2_	*f*	Cat (*n* = 1)	o	x
1982	East [32]	PID	PaCO_2_	*f*	Dogs (*n* = 18)	x	o
1982	Ohlson [30]	PID	etCO_2_	*V*_*T*_	Dogs (*n* = 6)	o	x
1984	Bhansali [33]	P	etCO_2_	*V*_*T*_	Dogs (*n* = 3)	x	x
1985	Chapman [34]	PI	etCO_2_	MV	Dogs (*n* = 5)	x	x
1987	Ritchie [35]	PI	etCO_2_	*V*_*T*_	Dogs (*n* = 5)	x	x
1994	Takahara[17]	Adaptive	etCO_2_	*V*_*T*_	Patients (*n* = 10)	x	o
1996	Schäublin [23]	Fuzzy	etCO_2_	*V*_*T*_, *f*	Patients (*n* = 30)	x	o
2002	Fernando [21]	MPC	PaCO_2_	MMV level	Patient (*n* = 1)	x	o
2004	Martinoni [22]	MPC	etCO_2_	MV	Patients (*n* = 15)	x	x

Setpoint control is the dynamic response of the system to changes of the target. Disturbance control is the response of the system to an external disturbance (e.g., extracorporeal CO_2_ loading, pulmonary artery occlusion or disconnection)

The focus was mostly on the control of CO_2_ until the early 1970s, but in 1975, Mitamura [36] developed a “dual control system” for both CO_2_ and O_2_. Here, the SpO_2_ was measured using an ear oximeter and an on-off controller was used for changing the inhaled oxygen content. The controller was able to rectify the hypoxia.

Controlling only the oxygenation was performed in preterm infants by Beddis et al. [37] in 1979; this was made possible by using an indwelling umbilical arterial oxygen electrode sensor. To evaluate their system, they compared the time spent at the oxygenation target using the controller to a clinician-in-the-loop system. This metric was also used by others [38–44] and the automated system was as good or better than the manual procedure in all cases.

Yu et al. later used an oximeter on the tongue to control the oxygenation and implemented an adaptive controller in dogs [19]. This system was able to rectify hypoxia and compensate for disturbances, such as positive end-expiratory pressure (PEEP) changes and one lung ventilation. In 1991, East et al. [45] used both FiO_2_ and PEEP to control PaO_2_. Whether to change FiO_2_ or PEEP was based on previous clinical protocols and the system kept the patients at the oxygenation target for up to 6 h [45].

Further literature on oxygenation control is summarized in Table 2. The control of oxygenation using the fraction of inspired oxygen remains an active field of research, especially in neonates. A recent review of oxygenation control was published by Claure and Bancalari in 2013 [46]. The systems presented by Claure et al. [41], Urschitz et al. [42], and Gajdos et al. [44] have been developed further and are now commercially available as AVEA-CLiO2 (CareFusion, Yorba Linda, CA, USA), CLAC (Löwenstein Medical GmbH & Co. KG, Bad Ems, Germany), and SPOC (Fritz Stephan GmbH, Gackenbach, Germany), respectively.

**Table 2:** Chronological development of closed-loop ventilation for O_2_ control in vivo

Year	First author	Controller type	Patient	Ventilation variables	Subject	Setpoint control	Compared to Manual
1975	Mitamura [36]	On/off	SaO_2_	FiO_2_	–	x	o
1979	Beddis [37]	P	PaO_2_	FiO_2_	Neonates (*n* = 12)	o	o
1985	Sano [18]	Adaptive	tcPO_2_	FiO_2_	Dogs (*n* = 2)	x	o
1987	Yu [19]	Adaptive	SpO_2_	FiO_2_	Dogs (*n* = 8)	x	o
1988	Dugdale [38]	Robust	PaO_2_	FiO_2_	Neonates *(n* = 7)	o	x
1991	East [45]	PID	PaO_2_	PEEP, FiO_2_	Dogs (*n* = 4)	x	o
1992	Bhutani [39]	PID	SaO_2_	FiO_2_	Neonates (*n* = 14)	o	x
1995	Waisel [40]	Expert	SaO_2_	FiO_2_, PEEP	Patients (*n* = 6)	o	x
1997	Raemer [47]	PID	SpO_2_	FiO_2_	Dogs (*n* = 6)	x	o
2001	Claure [41]	Rule-based	SpO_2_	FiO_2_	Neonates *(n* = 14)	o	x
2004	Urschitz [42]	Expert	SpO_2_	FiO_2_	Neonates (*n* = 12)	o	x
2008	Johannigman [48]	PID	SpO_2_	FiO_2_	Patients (*n* = 15)	x	o
2017	Morozoff [49]	Adaptive	SaO_2_	FiO_2_	Neonates (*n* = 7)	o	x
2018	Gajdos [44]	Adaptive	SpO_2_	FiO_2_	Neonates (*n* = 12)	o	x

Setpoint control is again the dynamic response of the system to changes of the target. Disturbance rejection was seldom evaluated for O_2_ control systems. Instead, the controller was compared to a manual (clinician-in-the-loop) system, with the metric being the total time spent at the target zone

Importantly, the control of oxygenation has mostly been limited to using only the FiO_2_ so far. This closely reflects the clinical difficulty in correctly choosing the PEEP—not least because oxygenation alone is not a reliable measurement of a good PEEP.

A large disadvantage of most of the closed-loop ventilation strategies presented is that they focus only on gas exchange and do not consider lung mechanics and quantification of harm. In fact, achieving proper CO_2_ control may require excessive *V*_*T*_ or peak inspiration pressure, which can cause ventilator-induced lung injury (VILI). With clinical ventilation strategies becoming focused on being protective and preventing VILI, this requirement also needed to be incorporated into closed-loop control. Hence, control considering lung mechanics is presented next.

### Control considering lung mechanics

Mitamura et al. considered minimizing ventilatory work as a further goal of their controller as early as 1971 [20]. The idea is closely related to that of Otis et al. [50] from 1950, which suggests that there exists an optimal combination of respiratory rate and tidal volume for minimal work of breathing. This approach was also used by Tehrani [51] and Laubscher [52] in 1991 and 1994, respectively. Laubscher et al. showed that their controller was able to adapt to personalized respiratory mechanics in a study on six patients. Laubscher and colleagues advanced their adaptive lung ventilation (ALV) controller (1994) to a newer version called *adaptive support ventilation* (ASV). Arnal et al. [53] tested the ASV controller on 243 patients with different respiratory lung conditions and showed the ability of the controller to choose *V*_*T*_– *f* combinations related to actual personalized lung mechanics.

Rudowski et al. [54] addressed the concerns of VILI directly with the peak respiratory power index, as an index of lung trauma, in 1991. Their controller adjusts ventilator settings to reduce the respiratory power index, while ensuring adequate gas exchange. A study with six patients showed promising results.

Many modern systems do not directly control the ventilator using lung mechanics, but rather apply hard limits to ensure that tidal volume and pressure stay within certain, evidence-based, limits.

### Control based on patient demand

It is also important to note here that the majority of literature presented so far considers only controlled (mandatory) ventilation, i.e., the patient is passive. However, in many cases, the patient is allowed or expected to breathe spontaneously, meaning patient demand becomes important. Early work was performed by Younes et al. with proportional assist ventilation (PAV) in 1992 [55]. This positive feedback control system amplified patient effort, according to the respiratory mechanics and level of assistance set by the operator. This ensures synchrony, while automatically adapting to patient load. For the initial version of PAV, the clinician required knowledge of the respiratory mechanics of the patient to set an appropriate controller gain, but the newer version, called PAV+, estimates the individual respiratory mechanics automatically [56, 57].

In 1996, Iotti et al. proposed *P*_0.1_ closed-loop control ventilation, whereby the drop in airway occlusion pressure during the first 0.1 s of inspiration is used to estimate patient work [58]. Two independent controllers, one for *P*_0.1_ and the other for alveolar volume, are fed into a merged control algorithm which changes the level of pressure support. The authors showed that inspiration activity of the patient can be stabilized at a desired level using *P*_0.1_, thus allowing for the unloading of the inspiratory muscles.

A direct coupling to the physiological neural output of the respiratory system would be helpful for optimal support during spontaneous breathing. An attempt to couple a respirator to phrenic nerve activity was performed in 1970 on animals [59], but this was not feasible in humans. Instead, Sinderby et al. [60] used the diaphragmatic electrical activity (EAdi) for neuro-ventilatory coupling to adjust the level of ventilatory support. This system requires the placement of an esophageal catheter. This system is commercially available as neurally adjusted ventilatory assist (NAVA) (Maquet Critical Care AB, Solna, Sweden). Improved patient-ventilator synchrony for this system was shown by [61], but the authors noted that the clinical impact thereof still needed to be determined.

### Automation of clinical protocols

There have been various approaches to computerize clinical protocols which medical staff use to adapt mechanical ventilator settings. These computerized decision support systems do not make active changes to the ventilator but rather propose a change; the clinician has to be present to make the change and, as such, they represent classical clinician-in-the-loop systems. A literature review of these systems is given in [62].

Pomprapa et al. automated the ARDSNet protocol (autoARDSNet) and tested the system in a pilot study with seven pigs [63]. The system kept the animals at the oxygenation and pH targets for a duration of 4 h, even compensating for a disconnection between ventilator and patient.

## Highly automated systems

**Fig. 4:**
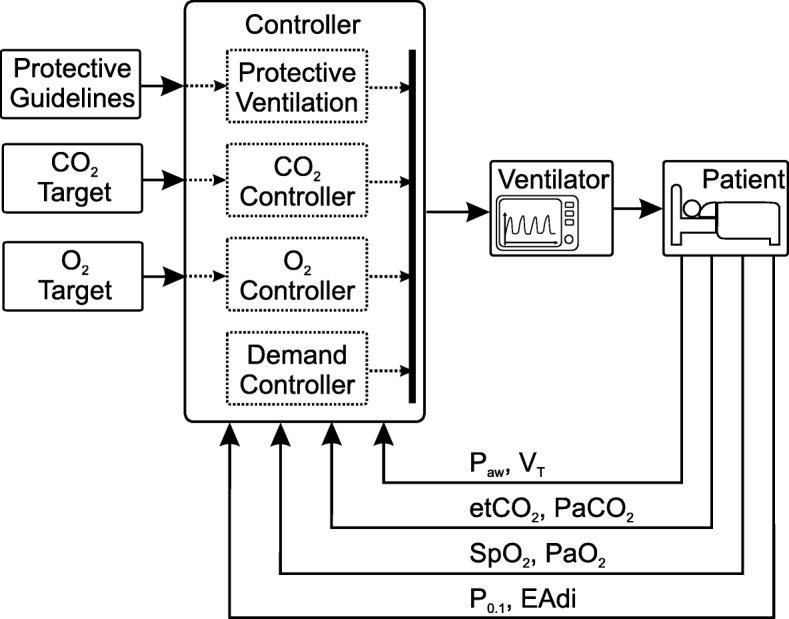
Control topology for a fully automated physiological closed-loop ventilation. Measurement signals fed back to the controller are categorized according to the control target. The list of physiological measurements is not complete but shows only examples taken from the presented PCLC systems

Academia and industry have started to present highly and even fully automated systems which rely on a culmination of all the sub-categories of control targets presented above. This involves keeping an acceptable homeostasis of blood gases, ensuring patient-ventilator synchrony and preventing VILI. The topology for such a control loop is shown in Fig. 4.

The commercially available INTELLiVENT$^{\circledR }$ - ASV$^{\circledR }$ (Hamilton Medical AG, Bonaduz, Switzerland) is one example of a highly automated system. The control targets are the etCO_2_ and SpO_2_. In addition, the user has to provide some patient data, which is used for the estimation of lung mechanics. Oxygenation control is achieved by changing either PEEP or FiO_2_, according to the clinical guidelines from the ARDSNet protocol. Ventilation control (CO_2_ elimination) is controlled by a cascaded control loop, which includes the ASV controller described above.

Another highly automated system has been presented by Schwaiberger et al. in 2018 [64]. The etCO_2_ was controlled using fuzzy control; the principles of the open lung concept [65] were used for improved oxygenation and low *V*_*T*_ ventilation ensuring protective ventilation was achieved [66]. A pilot study on eight pigs showed promising results but clinical trials are still pending. Furthermore, this system has only been applicable to passive patients.

## Weaning

The final phase of mechanical ventilation has already seen some of the most advanced closed-loop systems becoming commercially available. The same control targets presented above also hold true for the weaning phase, but an additional goal during weaning is to reduce the reliance of the patient on the ventilator and to test whether the patient can be taken off mechanical aid completely.

A first approach to automated weaning was made by Hewlett et al. in 1977 [67] with mandatory minute ventilation.

However, the first automated physiological weaning approach was proposed by Chopin et al. in 1989 [68]. In their “carbon dioxide mandatory ventilation (CO2MV)” method, the authors used etCO_2_ and a rule-based controller to switch between spontaneous and controlled ventilation. In 1991, Strickland and Hasson [69] used pulse oximetry, respiratory rate, and minute ventilation to automatically set the synchronized intermittent mandatory ventilation rate and level of pressure support ventilation (PSV). Weaning was considered complete when the pressure support had reached zero; two or less mandatory breaths per minute were required and BGA showed sufficient evidence.

Dojat et al. [70] developed the GANESH system, which used the etCO_2_, respiratory rate, and *V*_*T*_ as inputs for their rule-based controller to set the level of PSV. The goal of this strategy was to bring the patient into a *zone of comfort*, at which point permanent weaning was envisaged. This system was developed further, called NéoGanesh, and presented in 1997 [71] with a sophisticated knowledge and temporal reasoning controller. Finally, this system is available as SmartCare$^{\circledR }$/PS from Dräger (Drägerwerk AG, Lübeck, Germany).

The ALV controller by Laubscher et al. described previously was used for weaning by Linton and colleagues in 1994 [72]. Expanding on ALV, the ASV was developed. Given a patient capable of initiating spontaneous breathing, the ASV algorithm automatically decreases the inspiratory pressure. Once all breaths are spontaneous and stable gas exchange is ensured, weaning is considered complete. There was no direct feedback of gas exchange in the early versions. This has changed with the introduction of the INTELLiVENT$^{\circledR }$ - ASV$^{\circledR }$.

## Evaluation of commercial physiological closed-loop ventilation

The papers presented so far have shown novel methods of applying physiological closed-loop control to mechanical ventilation and were able to test the systems on medium/large animals or a small number of patients. The commercial availability of some of these systems has allowed clinical studies to be performed. Brogi et al. did a systematic review and meta-analysis of closed-loop systems in the clinical environment. Five studies using automated FiO_2_ adjustments based on SpO_2_ measurements and three studies investigating ventilation control (etCO_2_) were included in the review. The time spent in the target zone was longer for automated systems in all studies [73]. The NAVA system was evaluated in a study by Demoule et al. and compared to the ventilation with PSV. Patient-ventilator asynchrony was reduced despite not increasing the probability of remaining in a partial ventilatory mode [74]. Rose et al. did a Cochrane systematic review and meta-analysis of clinical trials comparing automated and non-automated weaning [75]. The review included 21 trials, totaling 1676 patients, and included automated systems such as SmartCare$^{\circledR }$/PS, ALV, and INTELLiVENT$^{\circledR }$ - ASV$^{\circledR }$. They found that the weaning and ventilation duration was significantly reduced by automated systems [75]. Importantly, no strong effect on mortality was found. Burns et al. used the SmartCare system to compare closed-loop control of weaning to the manual protocol-based weaning [76]. They concluded that automated weaning showed promising results, but warranted further investigation [76]. A randomized controlled study by Lellouche et al. showed that the required number of interventions by clinical staff was reduced when using a fully automated ventilation system, thus, reducing the workload of the staff [77]. Arnal et al. showed that the INTELLiVENT$^{\circledR }$ - ASV$^{\circledR }$ reduced the number of manual ventilator setting changes in ICU patients [6]. They further concluded that this may increase the efficiency of the workforce [6].

These first analyses of clinical studies show promise that the application of PCLC to mechanical ventilation can reduce the workload of clinical staff and keep patients within personalized oxygenation and ventilation target zones safely. However, whether fully automated system will lead to improved mortality rates remains an open question.

## Outlook

The evolution of the PCLC in mechanical ventilation continues to be driven by new controller designs, improved modeling, new sensor modalities, and better ventilation strategies.

Improved controller designs are needed that consider the cross-coupling effects of ventilator settings. Controlling CO2 by changing the MV will indirectly affect the oxygenation—which is not considered by any of the presented PCLC systems.

New measurements and imaging techniques are being applied in the “clinician-in-the-loop” setting, which could be used for automatic control in the future. The transpulmonary pressure, derived by subtracting the measured esophageal pressure from the airway pressure, is being researched as an approach to finding the appropriate PEEP setting [78]. Electrical impedance tomography (EIT), as a measurement technique, is gaining acceptance to titrate PEEP and also gain insight into the ventilated lungs. Recent reviews of electrical impedance tomography are presented by [79–81].

Derived evaluations, such as the oxygen A-a gradient for oxygenation or stress and strain for protective ventilation, need to be incorporated into PCLC systems. The hemodynamic effects of ventilator changes (e.g., increasing PEEP) also need to be accounted for by the PCLC system. Furthermore, the medical history of the patient should be considered by the PCLC system.

With most data becoming digitized, it is plausible that this data will be available to the ventilator in the future. In such a *cyber-medical* world, all data will be transferred between all medical devices and this data will allow artificial intelligence to be applied to mechanical ventilation. Prasad and colleagues described a decision support system based on artificial intelligence [82].

Despite the many advantages that come with PCLC, the dangers thereof may not be forgotten. Sensor failure, unpredictable disturbances and software errors remain issues in any automated system and safety concepts need to be developed to ensure no harm is done to the patient. Kuck and Johnson [83] formulate the three laws for automation in anesthesia nicely: (1) do no harm, (2) be transparent, and (3) reduce the cognitive workload. A similar approach should be taken in the further development of closed-loop control of mechanical ventilation.

## Conclusion

The application of PCLC to mechanical ventilation started well over half a century ago and all this research is beginning to bear fruit resulting in highly automated ventilators. As these first systems become commercially available, it is expected that more will follow, fed with new technology from industry and academia and, as such, the dawn of physiological closed-loop ventilation has certainly arrived.

## Data Availability

Not applicable.

## Abbreviations

ALV: Adaptive lung ventilation
ASV: Adaptive support ventilation
BGA: Blood gas analysis
CO_2_: Carbon dioxide
EAdi: Diaphragmatic electrical activity
EIT: Electrical impedance tomography
etCO_2_: End-tidal carbon dioxide
f: Breathing frequency
FiCO_2_: Fraction inspired carbon dioxide
FiO_2_: Fraction inspired oxygen
I: E: Inspiration to expiration ratio
MMV: Mandatory minute ventilation
MV: Minute ventilation
NAVA: Neurally adjusted ventilatory assist
*P*_insp_: Inspiratory pressure
PaCO_2_: Arterial carbon dioxide tension
PaO_2_: Arterial oxygen tension
PAV: Proportional assist ventilation
PCLC: Physiological closed-loop ventilation
PEEP: Positive end-expiratory pressure
PID: Proportional, integral, and differential
PIP: Peak inspiratory pressure
PSV: Pressure support ventilation
O_2_: Oxygen
SaO_2_: Arterial oxygen saturation
SIMV: Synchronized intermittent mandatory ventilation
SpO_2_: Oxygen saturation (measured by oximetry)
tcO_2_: Transcutaneous oxygen tension
*V*_*T*_: Tidal volume
VILI: Ventilator-induced lung injury
